# ATM Kinase-Dependent Regulation of Autophagy: A Key Player in Senescence?

**DOI:** 10.3389/fcell.2020.599048

**Published:** 2021-01-07

**Authors:** Venturina Stagni, Alessandra Ferri, Claudia Cirotti, Daniela Barilà

**Affiliations:** ^1^Institute of Molecular Biology and Pathology, National Research Council (CNR), Rome, Italy; ^2^Laboratory of Cell Signaling, Istituto di Ricovero e Cura a Carattere Scientifico (IRCCS) Fondazione Santa Lucia, Rome, Italy; ^3^Department of Biology, University of Rome Tor Vergata, Rome, Italy

**Keywords:** ATM kinase, autophagy, senescence, ataxia-telangiectasia, DDR

## Abstract

Increasing evidence suggests a strong interplay between autophagy and genomic stability. Recently, several papers have demonstrated a molecular connection between the DNA Damage Response (DDR) and autophagy and have explored how this link influences cell fate and the choice between apoptosis and senescence in response to different stimuli. The aberrant deregulation of this interplay is linked to the development of pathologies, including cancer and neurodegeneration. Ataxia-telangiectasia mutated kinase (ATM) is the product of a gene that is lost in Ataxia-Telangiectasia (A-T), a rare genetic disorder characterized by ataxia and cerebellar neurodegeneration, defects in the immune response, higher incidence of lymphoma development, and premature aging. Importantly, ATM kinase plays a central role in the DDR, and it can finely tune the balance between senescence and apoptosis: activated ATM promotes autophagy and in particular sustains the lysosomal-mitochondrial axis, which in turn promotes senescence and inhibits apoptosis. Therefore, ATM is the key factor that enables cells to escape apoptosis by entering senescence through modulation of autophagy. Importantly, unlike apoptotic cells, senescent cells are viable and have the ability to secrete proinflammatory and mitogenic factors, thus influencing the cellular environment. In this review we aim to summarize recent advances in the understanding of molecular mechanisms linking DDR and autophagy to senescence, pointing out the role of ATM kinase in these cellular responses. The significance of this regulation in the pathogenesis of Ataxia-Telangiectasia will be discussed.

## Introduction

Autophagy is a highly conserved catabolic pathway necessary for the maintenance of cellular homeostasis (Rubinsztein et al., 2011). Physiologically, autophagy acts as a quality control pathway that eliminates damaged proteins and organelles. Instead, under stress conditions, it could induce a programmed cell death called “autophagy-dependent cell death” (ADCD) (Rubinsztein et al., 2011). Among autophagic pathways, selective mitochondrial degradation (mitophagy) and selective peroxisome degradation (pexophagy), have emerged as important homeostatic mechanisms (Wang and Wang, 2019). Overall, the autophagic pathway deregulation appears to be related to many biologic processes as cancer, cardiovascular diseases, aging, and neurodegeneration, (Levine and Kroemer, 2008).

DNA damage response (DDR) is another essential pathway in the control of cellular homeostasis (Jackson and Bartek, 2009). In response to DNA damage, cells activate a highly conserved and complex kinase-based signaling network, to safeguard genomic integrity (Jackson and Bartek, 2009). The DDR pathway consists of a series of tightly regulated events, starting from the detection of DNA damage, accumulation of DNA repair factors at the site of damage, and finally physical repair of the lesion. When DNA repair is unsuccessful, the same DDR network that directs repair can induce senescence or cell death (d’Adda di Fagagna, 2008; Ribezzo et al., 2016). Deficiency of the DDR pathway underlies many human diseases, including developmental disorders, neurodegeneration, cancer, and immune disorders. Moreover, pharmacological inhibition of DDR is often used in cancer treatment (Helleday et al., 2008).

Accumulating evidence has demonstrated a strong connection between autophagy and DDR in the maintenance of cellular homeostasis (Eliopoulos et al., 2016). Autophagy acts as a source of energy during cell cycle arrest and during repair mechanisms, under DNA damage conditions (Eliopoulos et al., 2016). On the other hand, alterations in autophagy can enhance DNA damage and can promote the onset of neurodegenerative disorders as well as tumor development, highlighting the importance of the crosstalk between autophagy and DDR pathways in the maintenance of genomic stability (Eliopoulos et al., 2016). In particular, activation of autophagy during DDR seems to play an essential role in the outcome of senescence, disfavoring the apoptotic response (Herranz and Gil, 2018). Conversely, autophagy defects are linked to alteration of DDR, in particular in senescence response (Hewitt and Korolchuk, 2017).

Ataxia-telangiectasia mutated (ATM) protein, a 350 kDa evolutionarily conserved serine/threonine protein kinase, was first identified as a central player in the DDR pathway (Shiloh and Ziv, 2013). Now it is well known that ATM kinase is activated also by several stimuli different from DNA damage such as reactive oxygen species (ROS), reactive nitrogen species (RNS), and starvation (Shiloh, 2014). Consistently, recent evidence demonstrates critical cytoplasmic functions of ATM, in addition to the classical functions in the nucleus in response to DDR (Ditch and Paull, 2012). In the cytoplasm, ATM has been shown to be located in peroxisomes, mitochondria, and endosomes, and it participates in sensing oxidative stress and in regulating cell metabolism and autophagy (Lee and Paull, 2020). Consistently, among DDR kinases, the ATM protein kinase has a unique, intriguing connection to autophagy (Stagni et al., 2018; Liang et al., 2019). ATM regulates autophagy not only upon DDR induction but also in ROS-induced autophagy, mitophagy, and pexophagy (Stagni et al., 2018). Interestingly, accumulating evidence shows that ATM regulates cellular homeostasis upon DDR and ROS induction through the autophagy-senescence axis (Liang et al., 2019).

Here, we focus on the link between DDR and senescence, pointing out the role of ATM kinase in the modulation of autophagy as a bridging point between these cellular responses. The significance of the deregulation of this equilibrium in the pathogenesis of Ataxia Telangiectasia will also be discussed.

## Crosstalk Between DDR and Autophagy

As described above, it is well documented that autophagy is induced by DNA damage, and it is required for several functional outcomes of the DDR, such as DNA repair, senescence, and cytokine secretion (Hewitt and Korolchuk, 2017). On the other hand, alterations of autophagy have been shown to increase DNA damage and to promote cancer and neurodegenerative disease occurrence (Hewitt and Korolchuk, 2017).

On the molecular level, it is well demonstrated that the DDR can trigger a rapid early induction of autophagy mediated by posttranslational modifications (PTMs), such as phosphorylation, ubiquitination, and acetylation (McEwan and Dikic, 2011). Interestingly, recent papers also report a slower, later induction of autophagy by DDR, mediated by transcriptional or posttranscriptional programs (Chen et al., 2020).

The first molecular player to be discovered between the DDR and autophagy was p53 (White, 2016). p53 is a well-known actor in the DDR pathway, and its role in this pathway has been largely reviewed elsewhere (Shiloh and Ziv, 2013), so it will not be discussed in this review. Recently, it has been discovered that p53 has a dual function in the control of autophagy: it can either activate or repress autophagy (White, 2016), depending on p53’s subcellular localization (Maiuri et al., 2010). Nuclear p53 can induce autophagy through the inhibition of mTOR by transcriptional upregulation of targets such as AMPK, PTEN, and Sestrins (Tang et al., 2015). Conversely, cytoplasmic p53 may inhibit autophagy through activation of AMP-dependent kinase (AMPK) and the consequent activation of mTOR independently of its transcriptional activity (Tasdemir et al., 2008a,b). Autophagy, in turn, represses p53 levels and function, significantly promoting tumorigenesis (White et al., 2015). For example, in a mouse model of hereditary breast cancer, allelic loss of *Atg6/Beclin1* extends mouse survival and suppresses tumor development, only when p53 is functional (Huo et al., 2013).

Another important player of the DDR that is also strongly involved in autophagy regulation is ATM kinase. Activation of ATM after exposure to genotoxic and oxidizing agents causes the repression of mTORC1 and the subsequent induction of autophagy (Alexander et al., 2010a,b). Moreover, upon DNA damage, ATM phosphorylates PTEN, promoting its nuclear localization and inducing autophagy as well (Chen et al., 2015). In addition, ATM sustains autophagy in breast cancer stem cells as it can promote the expression of ATG4C mRNA and protein (Antonelli et al., 2017). Consistent with the positive role of ATM on autophagy induction, ATM phosphorylates CHK2 and promotes FOXK nuclear export, following DNA damage, as has been recently demonstrated. In the process, phosphorylation prevents the inhibitory effect of ATM on the expression of autophagic genes and provides a novel mechanism that activates transcription of ATGs (Chen et al., 2020).

Interestingly, it has been demonstrated that the activation of ATM in the cytosol plays an essential role in the regulation of the autophagic response as well (Stagni et al., 2018). ATM is activated in the cytosol by ROS and hypoxia and can modulate autophagy through multiple molecular mechanisms. For example, in hypoxic conditions ATM inhibits mTORC1 by the regulation of the hypoxia-inducible factor (HIF-1α) transcription factor (Cam et al., 2010), while in response to ROS ATM can regulate pexophagy, through the phosphorylation of Pex5 (Zhang et al., 2015), and mitophagy through the modulation of Beclin-1 (Valentin-Vega et al., 2012; Guo et al., 2020). Overall, this evidence suggests that cytosolic functions of ATM are more related to autophagy regulation than to the canonical nuclear role, but how the balance between nuclear and cytosolic ATM functions is regulated is still unknown.

While the regulation of autophagy by DDR proteins is well documented, the molecular mechanisms behind the regulation of DDR protein functions by autophagy still remain a matter of debate (Eliopoulos et al., 2016). Some evidence suggests that autophagy inhibits the DDR by eliminating misfolded protein and damaged organelles (like mitochondria and peroxisome) that could trigger DNA damage, genome instability, and metabolic stress (Eliopoulos et al., 2016). Consistently, autophagy-deficient cells accumulate ROS and mitochondrial dysfunction together with DNA replication stress (Gomes et al., 2017). Moreover, defective autophagy sensitizes cells to metabolic stress and increases DNA damage (Rabinowitz and White, 2010; Gomes et al., 2017). At the molecular level it has been shown that loss of autophagy increases proteasomal activity resulting in an enhanced degradation of checkpoint kinase 1 (CHK1), a key enzyme for homologous recombination (HR) and increased micronuclei and sub-G1 DNA, markers of diminished genomic integrity (Liu et al., 2015). In addition, autophagy impairment-dependent accumulation of p62 promotes a direct binding of p62 to DDR proteins to inhibit the recruitment of the DNA repair proteins (Wang et al., 2016). Although the detailed molecular mechanism involved in autophagy-dependent regulation of DDR proteins is still largely unknown, a strong crosstalk occurs between DDR signaling and autophagy in the regulation of cellular homeostasis.

## The Autophagy-Senescence Connection in the DDR: Role of ATM Kinase

The link between the DDR and senescence was first associated with replication exhaustion at the end of the cellular lifespan, a process called replicative senescence (d’Adda di Fagagna, 2008; Rossiello et al., 2014). Telomere shortening is sensed by the cells as a double strand of DNA breaks and thereby triggers the activation of the ATM-p53 axis to elicit cell-cycle arrest and to execute senescence. The same link was identified also during persistent oncogenic signaling that triggers a powerful senescence response, known as oncogene-induced senescence (OIS) (Herranz and Gil, 2018). Enforced DNA replication induced by oncogene activation results in DDR and ATM kinase activation followed by activation of senescence, which must be considered a barrier to transformation (Liu et al., 2018). Interestingly, persistent DDR signaling mediated by ATM activation has been reported to contribute also to the acquisition of a proinflammatory senescence-associated secretory phenotype (SASP). It has been recently demonstrated in a model of naturally aged mice that activation of the ATM-NEMO-NF-κB axis is necessary for senescence induction and SASP, which in turn elicits DDR and SASP activation also in neighboring cells, thereby creating a proinflammatory environment (Zhao et al., 2020). Genetic or pharmacological inhibition of ATM reduces the adverse effects of chronic DNA damage, impinging on cellular senescence, improving stem cell functionality and extending health span. Consistently, by using high-throughput screening (HTS), the ATM inhibitor KU-60019 has been identified as an inhibitor of senescence in normal aging cells, which shows the importance of ATM kinase as an essential modulator of senescence (Kang et al., 2017; Kuk et al., 2019).

It is clear that ATM kinase plays a central role in connecting DDR to autophagy and DDR to senescence response, but whether autophagy is connected to senescence through ATM kinase is still debated. It has been demonstrated that the ATM-autophagy axis is responsible for the induction of senescence and the protection of cells against apoptosis upon DDR induction by anti-cancer drugs (Beauvarlet et al., 2019). Indeed, ATM activation upon G-quadruplex ligands (G4L) treatment drives cells to senescence to prevent cell death through activation of the autophagic pathway, pointing out the importance of ATM kinase as a modulator of senescence response through the regulation of autophagy (Beauvarlet et al., 2019). Consistently, disruption of either ATM or autophagy following G4L treatment impairs the induction of senescence and drives cells to apoptotic cell death (Beauvarlet et al., 2019).

**FIGURE 1:**
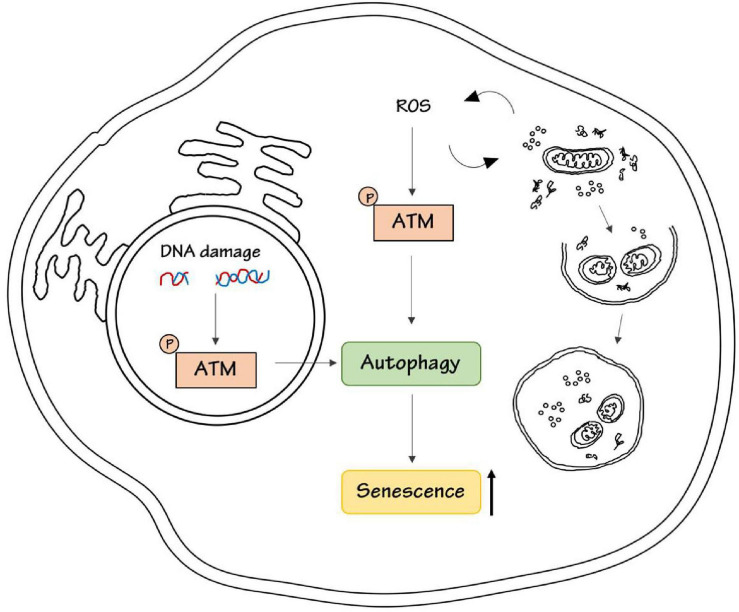
ATM kinase induces senescence. DNA damage or ROS stimulation activates ATM kinase respectively in the nucleus, to repair DNA damage, and in the cytoplasm, to maintain intracellular homeostasis by promoting the removal of damaged proteins and organelles. Both nuclear and cytoplasmic ATM has been shown to be involved in the regulation of autophagy either in a positive or negative way. ATM-dependent autophagy results in senescence induction.

Conversely, ATM was demonstrated to promote the acquisition of a senescent-associated secretory phenotype, SASP, by inhibiting the selective autophagy of the transcription factor GATA4 (Kang et al., 2015). Therefore, ATM could promote cell senescence through activation or inhibition of autophagy, probably depending on the cell type and on upstream stimuli (Figure 1).

Interestingly, another point of complexity in this regulation is that ATM kinase is essential for the regulation of autophagy not only upon DDR but also upon oxidative stress, as described above. The intracellular accumulation of oxidative damage triggered by ROS is considered a major determinant of senescence (Chandrasekaran et al., 2017). Recently it has been reported that ATM could be necessary in ROS-dependent senescence response induced by inhibitors of Vascular Endothelial Growth Factor (Mongiardi et al., 2019). Interestingly, autophagy is essential for the regulation of senescence upon ROS induction (Cordani et al., 2019). High ROS levels induce mitochondrial dysfunction and autophagy inhibition, which in turn promote cell senescence and generate vicious loop cycles in ROS production (Cordani et al., 2019). Given the central role of ATM in autophagy regulation upon ROS induction we could speculate that this ATM-autophagy axis could regulate senescence response also upon oxidative stress, but there is still no experimental evidence about this possible connection.

In conclusion, ATM kinase could link DNA damage and oxidative stress to autophagy, and this could be responsible for the senescence outcome (Figure 1).

## Conclusion

Ataxia-telangiectasia mutated is functionally inactivated in a genetic rare disorder called Ataxia-telangiectasia (A-T) (OMIM:208900). As we discussed above, it is clear that ATM kinase, by regulating autophagy, plays a central role in the induction of senescence and the suppression of apoptosis, both upon DNA damage and oxidative stress. What is the role of these pathways and of senescence in the development of the A-T pathology?

Ataxia-Telangiectasia (A-T) is an autosomal recessive disorder characterized by cancer susceptibility, radiation sensitivity, cerebellar degeneration, and telangiectasia (Shiloh and Ziv, 2013). Importantly, neurodegeneration and immune system defects in A-T have been regarded as a reflection of premature aging observed in A-T patients [reviewed in Shiloh and Lederman (2017)]. Evidence is growing that senescent cells accumulate during aging, promote chronic inflammation, and are associated with many age-related pathologies, including cancer and neurodegenerative disorders. Despite the fact that ATM kinase has been shown to promote senescence, the loss of ATM expression in A-T cells triggers a senescent-like phenotype as well (Table 1), including decreased replication capacity and shortening telomeres, as reported by several studies (Shiloh et al., 1982; Metcalfe et al., 1996; Xia et al., 1996; Smilenov et al., 1997; Wood et al., 2001; Naka et al., 2004; Shiloh and Lederman, 2017). In addition, A-T patients show increased levels of cytokines including IL-6 and IL-8 (McGrath-Morrow et al., 2016), elevated levels of Type I interferons (McGrath-Morrow et al., 2018), and chronic inflammation (Zaki-Dizaji et al., 2018). More recently, the upregulation of several genes associated with senescence and malignancy in A-T cells has been reported (McGrath-Morrow et al., 2020), consistently with the SASP phenotype and with premature senescence in A-T (Gatei et al., 2001; Stern et al., 2002; Weizman et al., 2003; Rashi-Elkeles et al., 2006; Shiloh and Lederman, 2017; Table 1). Interestingly, anti-inflammatory agents, such as betamethasone, can generate short-term improvement in A-T symptoms (Leuzzi et al., 2015; Hui et al., 2018).

**TABLE 1:** Summary of the key characteristics of senescent cells in A-T.

**Senescent phenotype in A-T**	**References**
Decreased replication	Shiloh et al., 1982; Metcalfe et al., 1996; Xia et al., 1996; Smilenov et al., 1997; Wood et al., 2001; Naka et al., 2004; Shiloh and Lederman, 2017
Telomeres shortening	Shiloh et al., 1982; Metcalfe et al., 1996; Xia et al., 1996; Smilenov et al., 1997; Wood et al., 2001; Naka et al., 2004; Shiloh and Lederman, 2017
Increased IL-6, IL-8	McGrath-Morrow et al., 2016
Elevated Type-I interferon levels	McGrath-Morrow et al., 2018
Chronic inflammation	Zaki-Dizaji et al., 2018
SASP phenotype	Gatei et al., 2001; Stern et al., 2002; Weizman et al., 2003; Rashi-Elkeles et al., 2006.

Why do A-T cells show a senescent phenotype? The apparent paradox between the role of ATM in the promotion of senescence and the senescent phenotype of A-T cells may be explained by the central role of ATM in the maintenance of cellular homeostasis upon different stress induction, as previously outlined.

The senescent phenotype could be explained in part by the fact that ATM-deficient tissues and cultured cells exhibit signs of chronic stress and low-level DDR that could contribute to SASP and senescence (Shiloh and Lederman, 2017; Sunderland et al., 2020). In addition, although A-T is considered a genome instability or DNA damage response syndrome and ATM is predominantly present in the nucleus of most mammalian cells, where it acts as an essential regulator of the DDR (Shiloh, 2014); several studies also supported the presence of ATM in the cytoplasmic compartment, including cytoplasmic vesicles, mitochondria, and peroxisomes, where ATM acts as a major regulator of proteostasis upon its oxidative stress-dependent cytosolic activation (Ditch and Paull, 2012; Lee and Paull, 2020). Given the role of ATM in the regulation of autophagy upon ROS induction (Stagni et al., 2018), we could hypothesize that not only persistent DNA damage, but also high ROS in A-T cells could contribute to premature senescence. The loss of ATM expression in A-T, resulting in an aberrant response to stress, dramatically breaks the ATM-autophagy axis and therefore unbalances this equilibrium, causing an accumulation of ROS, DNA damage, and senescence. These features may contribute to the overall loss of proteostasis and homeostasis control associated to this disorder (Lee and Paull, 2020).

The investigation of the molecular interconnection between ATM-autophagy and senescence will therefore support the comprehension of A-T pathogenesis and may also contribute to identify novel strategies to ameliorate patient’s management.

## Author Contributions

VS and DB: conceptualization. VS: writing – original draft preparation. DB, VS, AF, and CC: writing – review and editing. DB and VS: supervision. DB: funding acquisition. All authors have read and agreed to the published version of the manuscript.

## Conflict of Interest

The authors declare that the research was conducted in the absence of any commercial or financial relationships that could be construed as a potential conflict of interest.
